# Factors affecting the value revitalization of Qajar religious schools in Tehran

**DOI:** 10.1186/s40494-021-00526-z

**Published:** 2021-05-08

**Authors:** Mohammad Sadegh Taher Tolou Del, Bahram Saleh Sedghpour, Sina Kamali Tabrizi

**Affiliations:** 1 Faculty of Architectural Engineering and Urban Design, Shahid Rajaee Teacher Training University, Tehran, Iran; 2 Faculty of Humanities, Shahid Rajaee Teacher Training University, Tehran, Iran; 3 Faculty of Architectural Engineering, Shahid Rajaee Teacher Training University, Tehran, Iran

**Keywords:** Architectural heritage, Value-based conservation, Semantic values, Physical values, Delphi method

## Abstract

Nowadays, due to natural erosion and urban development, Qajar religious schools in Tehran have undergone adverse physical changes. Moreover, the semantic and intangible values of them have faded over time, such that their position in society has declined. The religious schools need the conservation and revitalization of their values. Various research has been conducted on the philosophy of education, and the spatial evolution history of Tehran’s religious schools. However, since no study has been carried out on the value revitalization of them, the present study, as an exploratory and novel study, mainly aims to experimentally investigate experts’ views to revitalize the value of Qajar religious schools in Tehran. Delphi research method and Q-type factor analysis were used to identify and classify experts’ views, respectively. Participants were selected through the purposive non-probability sampling technique. The sample size was selected to be 25, which was confirmed according to the Kaiser–Meyer–Olkin test used for sampling adequacy. To collect data from experts, a questionnaire was used in three rounds based on the Delphi method. Data obtained from the questionnaires were analyzed using the Q method. Based on the obtained results, up to 73.267% of factors affecting the value revitalization of Qajar religious schools in Tehran were identified and interpreted with certainty based on interviews with the experts. The experts were divided into eight groups or factors based on their views, and seven of which introduced common variables and concepts, named based on their constituent variables. Based on the value of the coefficient of variation, the identified effective factors included climatic architecture (20.51%), resilient architecture (13.45%), spiritual architecture (8.98%), environmental aesthetics (8.12%), educational architecture (6.87%), structural architecture (6.450%), and site visiting (4.566%). It was concluded that in the value revitalization of the religious schools, paying serious attention to these seven effective factors underlies the conservation process.

## Introduction

**Fig. 1:**
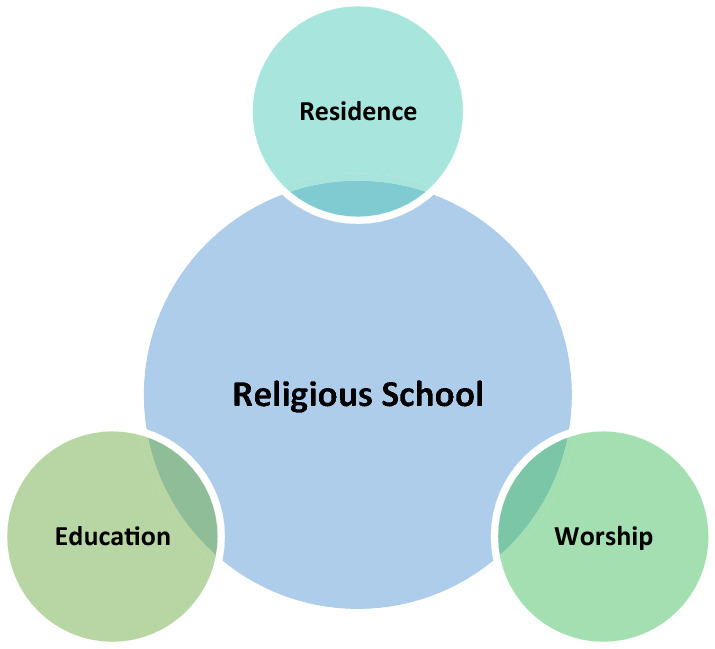
The combination of educational, worship, and residential spaces in the buildings of religious schools (Source: [3])

The school was an educational environment in Iran during the Islamic period. About the significance of learning, the Prophet of Islam says, learning is obligatory for every Muslim. A school can be briefly defined as an institution for higher education, in which traditional Islamic sciences such as hadith, Quranic exegesis, and religious issues are taught [1]. Religious sciences were taught in mosques and scholars’ houses in the early centuries. Gradually, with the expansion and advancement of religious sciences, the prolongation of education, and the need to accommodate students, grounds were prepared for the emergence of schools, most of which were located around mosques, markets, residential neighborhoods, squares, passages, and main streets and paths [2]. The main goal of religious schools was to educate students toward perfection using a combination of three spaces, namely, educational, worship, and residential spaces in one building (Fig. 1).

The emphasis on the teaching of religious sciences was always dependent on the ruling government at that time. The Qajar dynasty was one of the governments that had cared about the teaching of religious sciences [4], which is why many religious schools were built during this era. Meanwhile, Tehran, as the capital of Iran in the Qajar era, is the city with the greatest concentration of religious schools. During their 130 years of rule, 38 religious schools were built in Tehran, of which only 19 schools have been survived and not destroyed [5]. The schools are all located in the old areas of Tehran. Unfortunately, most of them have undergone inappropriate physical-semantic changes and their values have deteriorated. So, it is required to study them to develop a value-based conservation plan to prevent their demolition.

### Value-based conservation

In recent decades, international committees such as UNESCO and ICOMOS have been established to study and conserve cultural heritage, historical monuments, and sites. Based on international declarations, charters, and resolutions, attention to value-based conservation is of great importance (Table 1).

**Table 1:** Review of value-based conservation in international declarations, charters, and resolutions

Charter/year	Results
ICOMOS, 1964 (The Venice Charter)	The process of restoration is a highly specialized operation. Its aim is to preserve and reveal the aesthetic and historic value of the monument and is based on respect for original material and authentic documents [6]
UNESCO, 1972	This convention has emphasized outstanding universal values [7]
ICOMOS, 1983 (The Appleton Charter)	The better the values of cultural heritage are known and translated, the better its conservation and quality improvement will be [8]
ICOMOS, 1993 (10th General Assembly)	ICOMOS should respond much more as an international organization in situations where conservation values are at stake [9]
ICOMOS, 1994 (The Nara Document)	Conservation of cultural heritage in all its forms and historical periods is rooted in the values attributed to the heritage [10]
ICOMOS, 1999 (The Burra Charter)	Conservation of a place should identify and take into consideration all aspects of cultural and natural values without an unwarranted emphasis on one value at the expense of others [11]
Parks Canada, 2010 (The conservation standards in Canada)	Conservation practitioners operate in what is referred to as a ‘values-based context’ using a system that identifies and manages historic places according to values attributed through an evaluation process. These values generally include the aesthetic, historic, scientific, cultural, social, and/or spiritual importance of a place [12]
ICOMOS, 2010 (The New Zealand Charter)	Conservation of a place should be based on an understanding and appreciation of all aspects of its cultural heritage value, both tangible and intangible [13]

Conservation in architecture means protecting valuable architecture or architectural values and it is called value-based conservation [14]. The value-based approaches used in conservation possess have become dominant in academic and professional discourses from the early 1990s [15]. From this point of view, this issue has been emphasized under various titles: “The meanings and values of objects are the main reason for their conservation” [16], and “The reason for the conservation of objects by societies is that these things are valuable to people of those communities” [17] and “are preserved because they have values” [18]. In general, any conservation activity takes place when an object or place is valuable and therefore, decision-making on the treatment of the building and the intervention in it depends on these values [19]. Nowadays, value assessment plays the main role in architectural heritage conservation [19, 20]; as Fielden points out, the first step in the conservation process is to prioritize the values in the building [21]. About the prioritization of architectural heritage values, two general cases can be considered: 1. Works have one or two values and it is very easy to prioritize them; 2. Works have multiple and varied values and prioritizing values will become a necessity [14]. Values related to architectural heritage can be categorized into two main groups: tangible values (physical aspects), and intangible values (semantic aspects) [14, 22].

Conservation of the physical aspects related to the "profession and knowledge of the restoration" is a set of measures that rely on the improvement of tangible conditions, whether through a direct intervention leading to the manipulation of the physics and materials or through an indirect intervention leading to the manipulation of the surroundings or changing the influential factors of the historic building [21]. According to the New Zealand Charter, there are various degrees of intervention including (i) preservation, through stabilization, maintenance, or repair; (ii) restoration, through reassembly, reinstatement, or removal; (iii) reconstruction; and (iv) adaptation. Also, any intervention reducing or compromising the value of the architectural heritage is undesirable and should not occur [13].

According to the Nara Charter, conservation of the semantic aspects depends on the ability to identify, understand, and protect the intangible values [10]. Also, according to the Burra Charter, semantic conservation is a set of measures enabling a person to achieve the values, meanings, messages, and concepts latent in the heritage spaces [11].

In the value-based conservation process, effective values in the revitalization of the building should be identified by considering the function and use of the building [23]. Accordingly, this study mainly aimed to identify and examine experts’ views to revitalize the value of Qajar religious schools in Tehran City.

### Literature review

The studies done on religious schools are including the evolution of religious school spaces in different historical periods [24–32], the review of education history and educational topics and contents development [28–30, 32, 33], the review of the philosophy of education [28–30, 33], functional analysis [3, 34–36], spatial elements analysis [3, 5, 34], investigating the relationship between the mosque and school [37], investigating the yard effect in optimizing the ambient temperature of religious schools [38], the review of the ornaments of Qajar religious schools in Arak [39], the evaluation of Isfahan’s new religious schools built in the late Qajar period based on shaping factors and physical components [40], entrance typology based on the access way from the passageway to the mosque and school [35], and construction locating of religious schools [2].

### Religious schools spaces

**Fig. 2:**
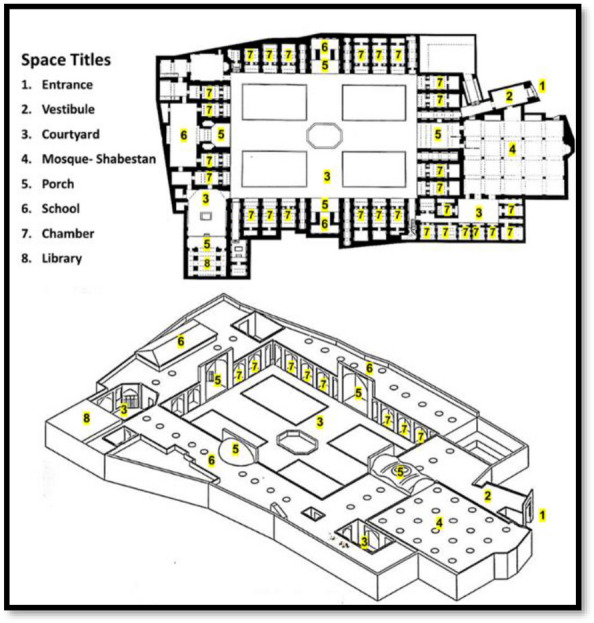
The introduction of spaces in the Marvi Religious School (Source: [41])

In terms of architecture, Qajar religious schools in Tehran have similarities and differences. Among these similarities are materials used in the buildings. Bricks were used in the construction of the schools. Generally, schools have one floor and, in some cases, two floors. Common spatial-functional elements in all these schools include the entrance space, the central courtyard, the chamber, the mosque (shabestan), the school, the porch, the wudu (ablution) room, and the bathroom (Fig. 2), which are mostly located around the central courtyard [5].

**Fig. 3:**
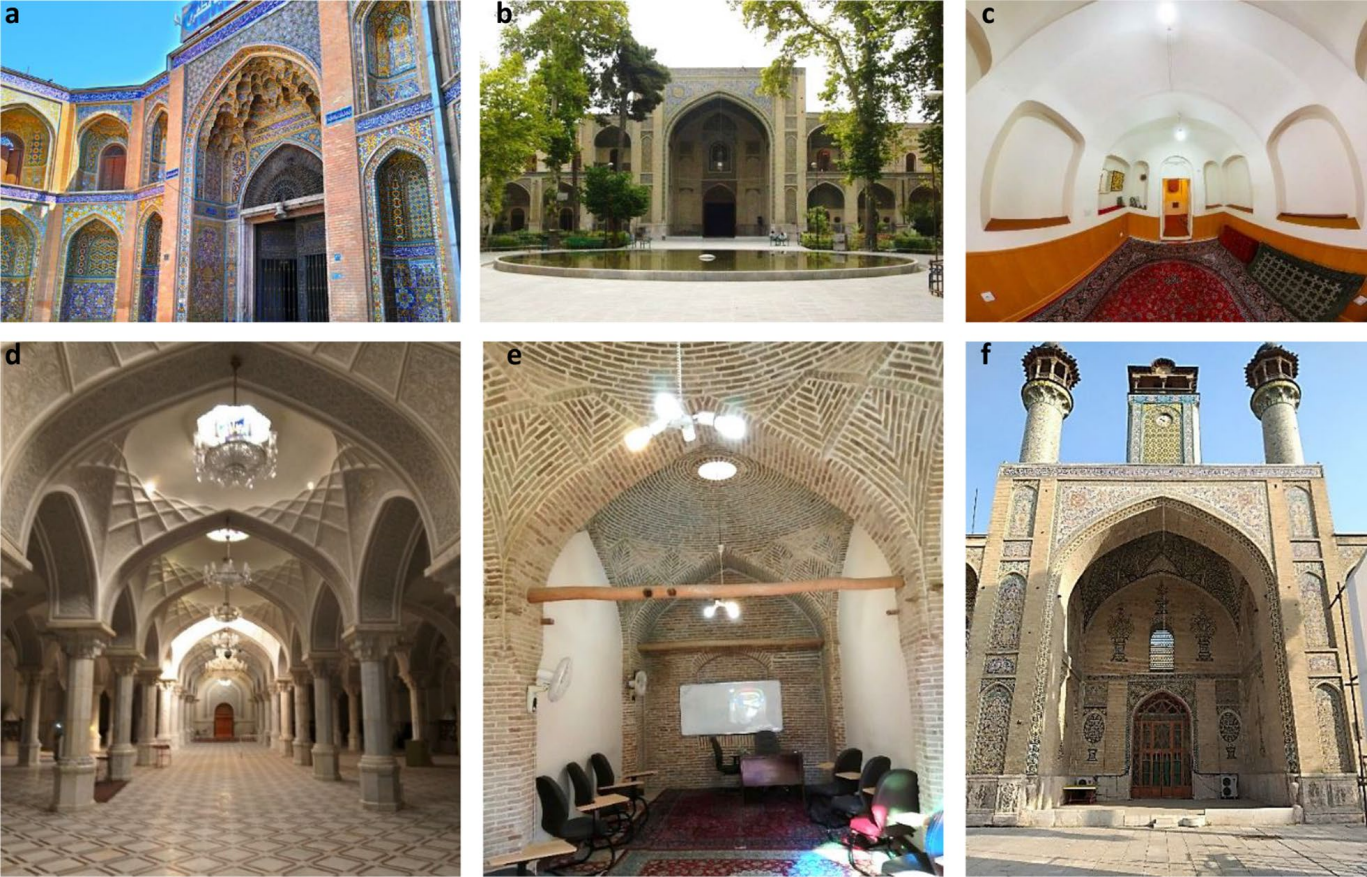
**a** The entrance space of the Sepahsalar, **b** The yard of the Sepahsalar, **c** The chamber of the Memarbashi (captured by fisheye lenses), **d** The mosque (shabestan) of the Sepahsalar, **e** The school of the Moayer Al-Mamalek, **f** The porch of the Sepahsalar

Briefly, the abovementioned elements are described as follows:

The entrance is a space with a tall and beautiful portal, that creates a sense of invitation to the religious school space [42]. The courtyard is a square or rectangular space called "Sahn" in mosques and religious buildings [43]. In religious schools, it has plants and a pond in its middle and it is a symbol of the introversion of Iranian architecture [42]. The chamber is a space, often with no specific geometry, that provided accommodation for students to stay and relax during studying. As one of the most characteristic spatial elements of a religious school, it plays the most significant role in forming the final shape of such schools [44] because each religious school had several chambers around the central courtyard. A mosque or shabestan is a place mainly used for worship. A mihrab is one of the elements in the shabestan architecture that indicates the qibla [5]. The school (madras) is a place where teachers teach their students, one or more of which are in each religious school [45]. The porch is a rectangular hall built at each side of a yard or courtyard and perpendicular to it, and its number has increased from one to two and four over time [46]. It provides a shady space for students to use in hot weather. The wudu (ablution) room is a place to perform ablutions before prayers. Wudu is a type of ritual purification or ablution in Islam (Fig. 3).

## Methodology

**Fig. 4:**
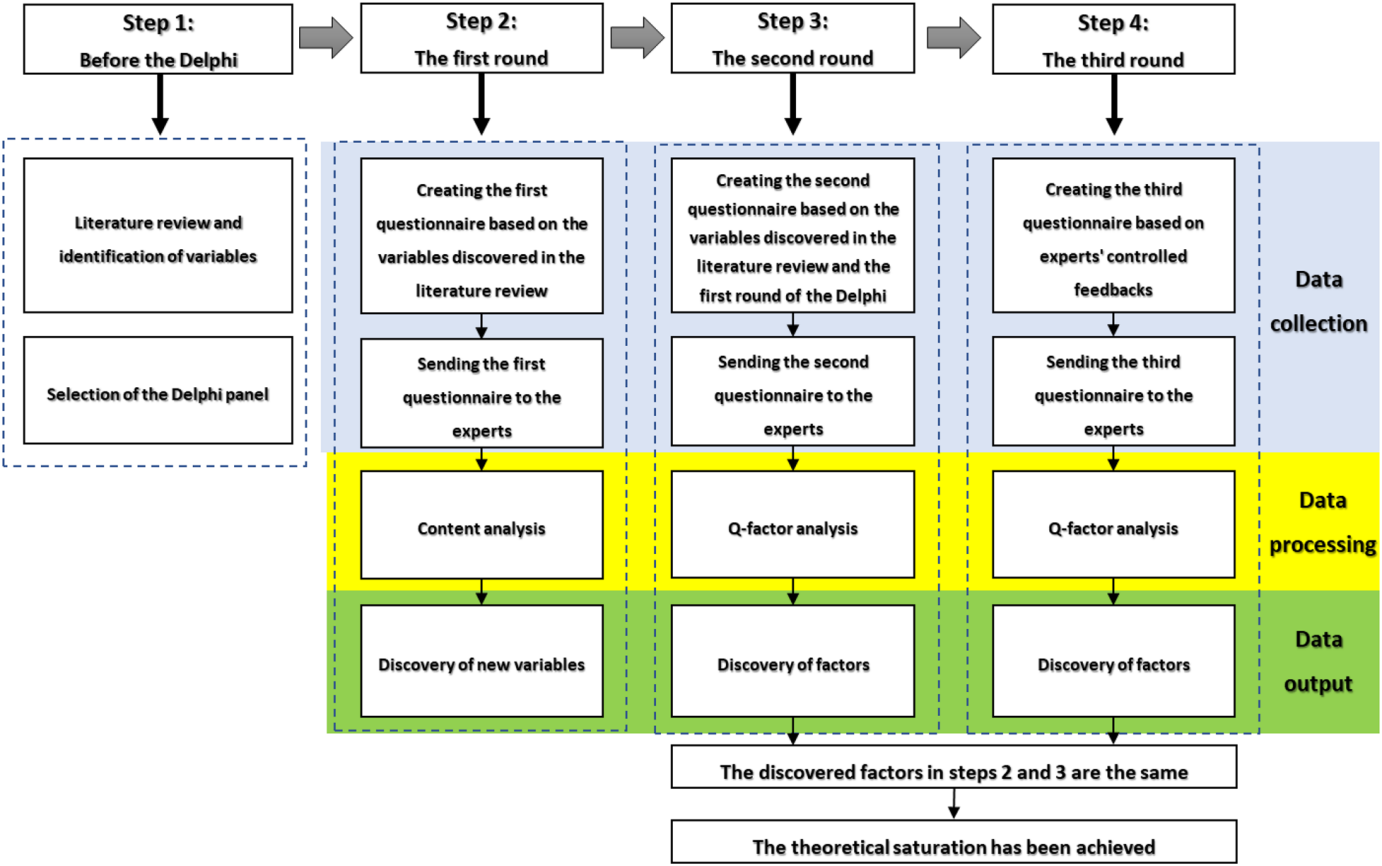
The methodology steps

The present study is exploratory due to the lack of theoretical foundations to identify factors affecting the value revitalization of religious-educational buildings. The Delphi method and the Q-type factor analysis were used to identify and classify experts' views [47–50], respectively (Fig. 4).

### The Delphi method

The Delphi method is a systematic research approach or method to extract opinions from a group of experts on a topic or question [51]. Many researchers [52–54] have used the definition proposed by Linstone and Turoff, where they define the Delphi method as a way to structure a group communication process so that it allows a group of individuals, as a whole, to solve a complex problem [55]. The main purpose of this method is to achieve the most reliable theoretical saturation of experts through a series of centralized questionnaires, along with controlled feedback [56]. Theoretical saturation means to reach a consensus on a viewpoint and to attempt to identify intellectual differences [57, 58].

*Experts' qualification process*: The Delphi method is performed with the participation of people who have knowledge and expertise in the research topic. These people are known as the Delphi panel [57]. Delphi participants are experts or panelists [57] with four characteristics as follows: (a) sufficient knowledge and experience in the subject, (b) willingness to cooperate, (c) sufficient time to participate in the rounds, and (d) effective communication skills [59–61]. The study experts are selected from the researchers of previous studies on religious schools, so they have specific mindsets and expertise based on the findings of their previous studies [62]. The number of Delphi participants is usually less than 50 and often 10 to 20 [57, 59, 63]. Therefore, the number of experts in this study is selected to be 25. After identifying the experts of historical religious schools from their published articles, they were invited to participate in this research through an e-mail. Due to the communication limitations of the Coronavirus, the authors had to communicate with experts through electronic questionnaires in the Delphi rounds.

*Case studies*: As mentioned before, during the Qajar era, 38 religious schools were built in Tehran, of which only 19 schools have been survived and not destroyed. So, in the present study, case studies are all 19 survived religious schools in Tehran namely, Sadr School, Khan Marvi, Hakim Bashi (Agha Mahmoud), Haj Rajabali, Abdollah Khan, Sheikh Abdol Hossein, The old Sepahsalar (Shahid Beheshti), Haj Ghanbar Ali Khan, Moayer Al-Mamalek, Khazen al-Molk, The new Sepahsalar (Shahid Motahari), Kazemieh, Memarbashi, Majd al-Doleh, Aqsa (Moshir al-Saltanah), Philsof al-Dowleh, Mahmudiya, Nezam al-Dowleh, and Mo'izz al-Dowleh.

### The Delphi features

Delphi is a series of surveys or interconnected questionnaires. The questionnaire in each round forms the next round questionnaire [64]. This technique has four fixed features as follows [65–67]:

1. The anonymity of participating members
2. Controlled feedback
3. The iteration of research steps
4. Statistical group response.

Anonymity refers to the anonymousness of participants or at least their responses in the Delphi method [68]. It allows each panel member to express their views and ideas without stress and being recognized by other members [55, 69, 70]. Controlled feedback refers to the opportunity for each expert to review their own and other experts' opinions in each round [71]. However, the exchange of information is not allowed between experts [57, 72]. It is a major part of reaching theoretical saturation [71, 73]. Iteration of steps refers to the iteration of a series of procedural, systematic, and written steps by a questionnaire to reach theoretical saturation [68]. According to previous studies, it is usually 2 to 10 steps [74]. However, according to Fan and Cheng (2006), three steps are sufficient for the Delphi method because the results are iterated in the second and third rounds [75]. Statistical group response refers to the statistical analysis of the data obtained from the Delphi rounds and group responses.

### The Delphi rounds

#### The first round

In the first round, a semi-structured questionnaire was administered, acting as a brainstorming strategy to reveal all issues related to the topic under study [68, 76].

After collecting the returned questionnaires, the responses were organized and shortened as much as possible, similar comments were combined and grouped, and repetitive topics were removed [61, 77]. The first-round responses were analyzed based on the research paradigm (qualitative codes and statistical summaries). This process is called content analysis [59]. The result of this round is discovered variables to develop a structured questionnaire that is used in the second round [78].

#### The second round

From the second round onwards, structured questionnaires were used and the participants were asked to quantify each variable using a Likert scale [79, 80]. To assess the mindset of the participants in the second and third rounds, a nine-point Likert scale questionnaire was designed and implemented. As the number 1 indicates the most disagreement, the number 9 indicates the most agreement, and the number 5 indicates the state of neutrality or hesitation with that variable [81]. After collecting the questionnaires in the second and third rounds, the obtained data were analyzed using Q-analysis. Then, the experts were classified based on their differences and commonalities with each other [82, 83]. Q-analysis is a technique that enables a researcher to identify and categorize individuals’ perceptions and beliefs based on their commonalities and differences [84]. The main purpose of this technique is to reveal different patterns of thinking, not to count the number of people with different thoughts. The Q study seeks to discover different mental patterns. To discover a pattern, the existence of only one person with that particular pattern is sufficient. In other words, the Q study does not show the "distribution" of individuals in different mental patterns to prove its claim by introducing a sample from the statistical community. However, it seeks to "prove the existence" of mental patterns. The Q study states that there are mental patterns of types A, B, and C. However, it does not mention the proportion of society with each pattern [85]. Factor analysis is the main statistical method for Q-analysis. This method functions by calculating the correlation coefficient between individuals. Hence, the term "Q-factor analysis" is used to emphasize that instead of variables, experts are categorized in the process of factor analysis. Nevertheless, statistically, there is no difference between Q-factor analysis and normal factor analysis. The process of Q-factor analysis, like exploratory factor analysis, consists of two steps: extracting (finding) factors as the first step and rotating them so that they can be interpreted. The principal components method is one of the most common methods of factor extraction to perform the first step of Q-factor analysis. The Varimax method is also a common method of factor rotation that has been used for data interpretability [86].

#### The third round

In the third round, participants were asked to review the responses (controlled feedback) and, if necessary, reconsider their opinions and judgments [87]. Typically, if responses obtained in this round are the same as those in the previous Delphi round, the iteration process is complete. Otherwise, the process is repeated until theoretical saturation is reached [70]. According to the similar results obtained in the second and third rounds of this study, there was no need to re-submit the questionnaire in the fourth round.

After completing the Delphi process and Q-factor analysis, i.e. the extraction and rotation of the factors, they were labeled and accurately interpreted. To interpret each factor, one should only pay attention to variables with very high or low scores that are common to the participants of each factor, because they represent ideas with great intensity. The identified variables were classified into two categories: discriminating or distinguishing variables and consensus variables. To name and interpret each factor, the main focus was on the distinguishing variables, i.e., variables causing two factors to be separated and considered different. In fact, such variables give identity to a factor and determine its specific meaning and concept. It is reasonable to expect a distinctive variable to receive different scores from different factors. In contrast to such a variable, there is a consensus variable with no significant difference between its points among different factors. To label and interpret each factor, after considering distinguishing variables, consensus variables must also be considered [88, 89].

## Results

Based on the two articles, a total of 74 physical [22] and semantic [14] variables were identified and given to the experts in the first Delphi round. As a result of the first round, in addition to the previous variables, three variables of artistic values, sense of belonging to the building, and compatibility values were discovered as new variables and added to the list of variables. Finally, based on the 77 variables, a Delphi round-two questionnaire was designed and implemented. Based on the Q-factor analysis on the data of the round-two questionnaire, seven factors were identified. The number of common variables detected by the experts with very high or low scores was 43, and 34 variables were removed in the second Delphi round. Then, the results were reported to the experts for controlled feedback. Based on the 43 variables, a Delphi round-three questionnaire was designed and implemented. In this round, data obtained from the questionnaire again underwent Q-factor analysis. This time, the experts identified eight factors, of which only the first seven had 34 common variables with very high or low scores, and thus, the eighth factor was removed. In other words, in the eighth factor, the score of the common variables was close to that of the neutral variables. The seven factors identified in the second and third rounds showed a common theme, and thus, there was no need to implement the fourth Delphi round.

To test the sampling adequacy, the sample adequacy index of Kaiser–Meyer–Olkin (KMO) was used. If the index value is above 0.6, the adequacy of the sample size is confirmed [90]. According to the obtained KMO value equal to 0.652, the adequacy of the research sample size was confirmed.

**Fig. 5:**
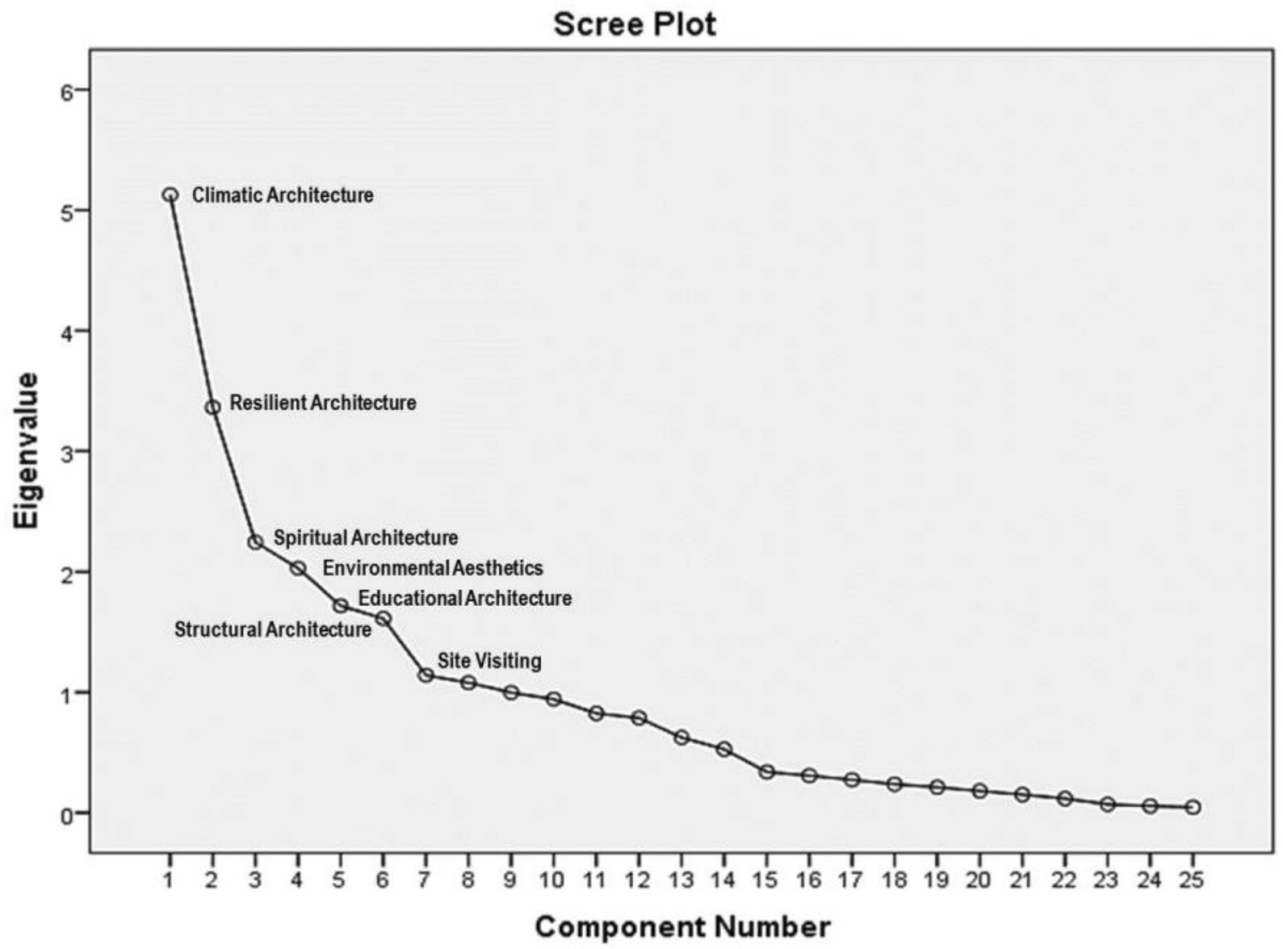
Results of Q-factor analysis in the Scree Plot chart

The results of Q-factor analysis can be observed in the Scree Plot chart (Fig. 5). All the eight factors had initial eigenvalues above one and were statistically significant. As mentioned, only seven of the factors were acceptable due to the existence of the common variables with very high or low scores. These seven identified factors are the same as the seven schools of thought. In other words, the experts’ opinions were grouped into seven distinct and interpretable categories.

Based on the results obtained in Table 2, up to 73.3% of the factors effective in revitalizing the value of Qajar religious schools in Tehran were identified and interpreted with certainty. The most significant school of thought was the first group of experts (the first factor), including 20.5% of the total variance.

**Table 2:** Total variance explained

Component	Initial Eigenvalues			Extraction sums of squared loadings		
	Total	% of Variance	Cumulative %	Total	% of Variance	Cumulative %
1	5.128	20.512	20.512	5.128	*20.512*	20.512
2	3.362	13.449	33.961	3.362	*13.449*	33.961
3	2.246	8.983	42.944	2.246	*8.983*	42.944
4	2.030	8.118	51.062	2.030	*8.118*	51.062
5	1.718	6.871	57.933	1.718	*6.871*	57.933
6	1.612	6.450	64.383	1.612	*6.450*	64.383
7	1.142	4.566	68.949	1.142	*4.566*	68.949
8	1.079	4.318	73.267	1.079	*4.318*	*73.267*
9	.997	3.988	77.254			
10	.942	3.768	81.022			
11	.825	3.299	84.321			
12	.787	3.147	87.468			
13	.624	2.497	89.965			
14	.527	2.107	92.072			
15	.338	1.353	93.425			
16	.308	1.231	94.656			
17	.273	1.092	95.747			
18	.236	.944	96.691			
19	.211	.845	97.536			
20	.180	.722	98.257			
21	.150	.600	98.857			
22	.117	.468	99.325			
23	.069	.275	99.600			
24	.055	.220	99.821			
25	.045	.179	100.000			

Italic values indicate significance of initial eigenvalues (total > 1)

To rotate the factors, the Varimax method was used. It is the common method to rotate factors for data interpretability. Based on the degree of the correlation coefficient among the experts, each factor was identified (Table 3). For example, regarding the first factor, five experts with numbers 18, 5, 9, 22, and 15 and regarding the second factor, three experts with numbers 2, 25, and 19 had similar opinions. The opinions of them were considered the first and second factors, respectively.

**Table 3:** Rotated component matrix^a^

Expert No	Component							
	1	2	3	4	5	6	7	8
Expert 18	*.843*	.246	.121	− .194	.001	.030	− .039	.230
Expert 5	*.822*	− .075	− .287	.137	.092	.020	.095	− .093
Expert 9	*.759*	.152	− .152	.029	− .055	.345	.011	.175
Expert 22	*.601*	.410	.212	.179	− .057	− .275	.114	.016
Expert 15	*.519*	− .013	.259	− .438	− .382	.104	− .158	− .150
Expert 2	− .061	*.873*	.159	− .019	− .012	.113	.046	− .052
Expert 25	.297	*.772*	.059	− .016	.142	− .233	− .040	.142
Expert 19	.284	*.767*	.076	.250	− .089	.180	.208	− .013
Expert 8	− .208	− .016	*.697*	.037	− .101	.081	.076	− .051
Expert 21	− .138	.358	*.690*	.255	.335	− .095	− .033	.012
Expert 3	.473	.053	*.628*	.146	− .121	− .139	.109	− .166
Expert 10	.409	.295	*.587*	.030	.329	− .231	.033	.246
Expert 17	.323	.197	*.400*	.234	.265	.322	− .146	.167
Expert 7	.063	.003	.175	*.811*	− .260	.095	.099	.014
Expert 4	− .104	.148	.218	*.692*	.202	.259	.216	.066
Expert 6	.275	.056	− .020	*.636*	.196	.038	− .561	.060
Expert 20	.127	.058	− .083	− .021	*.825*	.082	.201	− .091
Expert 14	.190	.248	− .203	.147	− *.561*	− .097	.319	− .190
Expert 24	− .215	.158	.380	.276	*.536*	− .148	.237	.331
Expert 13	.036	.248	− .021	.261	− .190	*.754*	.097	.000
Expert 16	− .093	.185	.042	− .015	− .235	− *.701*	.166	− .103
Expert 1	.204	.044	.069	.171	.229	− .177	*.778*	− .154
Expert 11	− .055	.225	.121	.123	.058	.210	*.528*	.437
Expert 12	.134	− .081	− .025	.111	.091	.031	− .046	*.841*
Expert 23	− .096	− .328	.150	.263	.273	− .134	.138	− *.433*

Italic values indicate a high correlation coefficient among the experts in each factor

Extraction Method: Principal Component Analysis

^a^Rotation Method: Varimax with Kaiser Normalization

After the identification of each factor by experts (Table 3), the authors referred to their responses. According to their responses, common variables with very high or low scores were identified in each factor, as presented in Table 4. Then, each factor was labeled and interpreted by considering the discriminating/distinguishing and consensus variables.

**Table 4:** Common variables with very high or low scores

No	1st Factor	2nd Factor	3rd Factor	4th Factor	5th Factor	6th Factor	7th Factor
	Climatic architecture	Resilient architecture	Spiritual architecture	Environmental aesthetics	Educational architecture	Structural architecture	Site visiting
1	Building proportions	Building proportions	Building proportions	Building proportions	–	–	–
2	Skyline	Skyline	–	–	–	–	Skyline
3	Plan design	Plan design	–	–	–	–	–
4	Roof Type	–	–	–	–	–	–
5	Structural system	Structural system	–	–	–	Structural system	–
6	–	Columns and bases		–	–	–	–
7	Building facades	Building facades	Building facades	–	–	–	–
8	–	–	–	–	–	–	Windows and openings
9	–	–	–	Yard and campus	–	–	Yard and campus
10	Architectural ornamentation	–	–	–	Architectural ornamentation	Architectural ornamentation	Architectural ornamentation
11	Vault and ceilings	Vault and ceilings	–	–	–	–	Vault and ceilings
12	Temperature and humidity	Temperature and humidity	–	–	–	–	–
13	–	Lighting	–	–	Lighting	–	–
14	Building form	Building form	Building form	–	–	–	–
15	Identity value	Identity value	Identity value	Identity value	Identity value	Identity value	Identity value
16	–	Cultural value	Cultural value	–	Cultural value	–	Cultural value
17	–	Place value	–	–	Place value	–	Place value
18	Historical value	Historical value	Historical value	–	–	Historical value	Historical value
19	–	Aesthetic value	Aesthetic value	Aesthetic Value	–	–	–
20	Integrity value	–	Integrity value	–	–	–	Integrity value
21	Authenticity value	Authenticity value	Authenticity value	–	Authenticity value	–	Authenticity value
22	–	–	Spiritual value	–	–	–	–
23	Architecture value	Architecture value	Architecture value	Architecture Value	Architecture value	Architecture value	–
24	–	Symbolic value	–	–	–	–	–
25	–	–	–	–	–	–	World registration
26	–	Social interaction value	–	–	Social Interaction value	–	–
27	–	Grandeur value	–	–	–	–	–
28	–	–	–	–	Educational value	–	–
29	–	Landscape value	–	Landscape value	Landscape value	–	–
30	–	Resilience value	–	–	–	–	–
31	–	Sense of belonging	–	Sense of belonging	Sense of belonging	Sense of belonging	–
32	–	Compatibility value	–	–	–	–	–
33	–	Artistic value	Artistic value	Artistic value	–	–	–
34	–	–	–	–	–	Economic value	–

*The first factor* (*climatic architecture*): Variables including the plan design, proportions, skyline, roof and covering, physical form, facade design, architectural ornamentation, and historical, architectural, and integrity values were related to architectural concepts. Moreover, the temperature and humidity of interior space, building structure type, the type of vault and ceilings of spaces, the identity value and authenticity of each building according to the climatic conditions of the region were related to climatic concepts. Thus, the first factor was related to the concepts of climatic architecture. In the religious schools of the Qajar era, the architecture of the buildings was compatible with the climate of the region (Table 5).

**Table 5:** Climatic architecture variables

Climatic architecture														
Architectural variables										Climatic variables				
Plan design	Building proportions	Skyline	Roof type	Building form	Building facades	Architectural ornamentation	Historical value	Architecture value	Integrity value	Temperature and Humidity	Structural system	Vault and ceilings	Identity value	Authenticity value

*The second factor* (*resilient architecture*): Variables including the plan design, proportions, skyline, facade design, physical form, and artistic, aesthetic, place, and architectural values were related to architectural concepts. Moreover, the sense of belonging to the building and the resilience, compatibility, social interaction, grandeur, landscape, symbolic, cultural, authenticity, identity, and historical values were considered the semantic resilience whereas the building structural system, pillars, and bases, vaults and ceilings, temperature, humidity, and lighting were regarded as the physical resilience. Thus, the second factor was related to the concepts of resilient architecture (Table 6). According to Alberti et al., resilience refers to "the degree to which a system is able to absorb risks and reorganize itself. Accordingly, resilience is a combination of absorbing disturbances and reaching a balance, self-reorganizing and increasing compatibility capacity" [91].

**Table 6:** Resilient architecture variables

Resilient architecture																								
Architectural variables									Resilience variables															
									Semantic resilience variables											Physical resilience variables				
Plan design	Building proportions	Skyline	Building facades	Artistic value	Aesthetic value	Building form	Place value	Architecture value	Resilience value	Compatibility value	Sense of Belonging	Social interaction value	Grandeur value	Landscape value	Symbolic value	Cultural value	Authenticity value	Identity value	Historical value	Structural system	Columns and bases	Vault and ceilings	Temperature and humidity	Lighting

*The third factor* (*spiritual architecture*): Variables including proportions, facade design, and physical form, and architectural, historical, integrity, authenticity, aesthetic, and artistic values were related to building architecture concepts. Moreover, the identity, cultural, and spiritual values, considering the religious use of these buildings, were related to spiritual concepts. Thus, the third factor was related to the concepts of spiritual architecture. In other words, spiritual concepts were expressed in the building through architecture (Table 7).

**Table 7:** Spiritual architecture variables

Spiritual architecture											
Architectural variables									Spiritual variables		
Building Proportions	Building facades	Building form	Architecture value	Historical value	Integrity value	Authenticity value	Aesthetic value	Artistic value	Identity value	Cultural value	Spiritual value

*The fourth factor* (*environmental aesthetics*): Variables including the yard and campus, sense of belonging, and architectural, and identity values were related to environmental concepts. Furthermore, the aesthetic, artistic, and landscape values and proportions were related to aesthetic concepts. As a result, the fourth factor was related to environmental aesthetic concepts (Table 8).

**Table 8:** Environmental aesthetics variables

Environmental aesthetics							
Environmental variables				Variables aesthetics			
Yard and campus	Architecture value	Sense of belonging	Identity value	Aesthetic value	Artistic value	Landscape value	Building proportions

*The fifth factor* (*educational architecture*): Variables including architectural ornamentation, lighting, sense of belonging, and place, architectural, and landscape values were related to architectural concepts. Moreover, the identity, cultural, authenticity, social interaction, and educational values were related to educational concepts. As a result, the fifth factor was related to the concepts of educational architecture. In fact, one of the main purposes of religious-educational buildings is to educate students through the language of architecture (Table 9).

**Table 9:** Educational architecture variables

Educational architecture										
Architectural variables						Educational variables				
Architectural ornamentation	Lighting	Place value	Architecture value	Landscape value	Sense of belonging	Identity value	Cultural value	Authenticity value	Social interaction value	Educational value

*The sixth factor* (*structural architecture*): Variables including the architectural ornamentation and value of the building were related to architectural concepts. Moreover, the structural system, sense of belonging, and identity, historical, and economic values were related to structural concepts. As a result, the sixth factor was related to the concepts of structural architecture. In fact, before the industrial revolution, in the design process, the building structure and architecture were considered and implemented together. In other words, architecture and structure were not separated (Table 10). As Marcus Vitruvius Pollio stated in 40 BC, "all buildings should have three attributes: firmitas, utilitas, and venustas, meaning: strength, utility, and beauty". Architecture should be beautiful and useful and its strength should be provided by a suitable structure [92].

**Table 10:** Structural architecture variables

Structural architecture						
Architectural variables		Structural variables				
Architectural ornamentation	Architecture value	Structural system	Identity value	Historical value	Sense of belonging	Economic value

*The seventh factor* (*site visiting*): Variables including the yard and campus, and identity, place, cultural, historical, integrity, and authenticity values were related to site concepts. Additionally, skyline, building windows and openings, building architectural ornamentations, vaults and ceilings of spaces, and world registration were related to visiting concepts. As a result, the seventh factor was related to the concepts of site visiting (Table 11).

**Table 11:** Site visiting variables

Site visiting											
Site variables							Visiting variables				
Yard and campus	Identity value	Cultural value	Place value	Historical value	Integrity value	Authenticity value	Skyline	Windows and openings	Architectural ornamentation	Vault and ceilings	World registration

## Discussion

### Summary of major findings

**Fig. 6:**
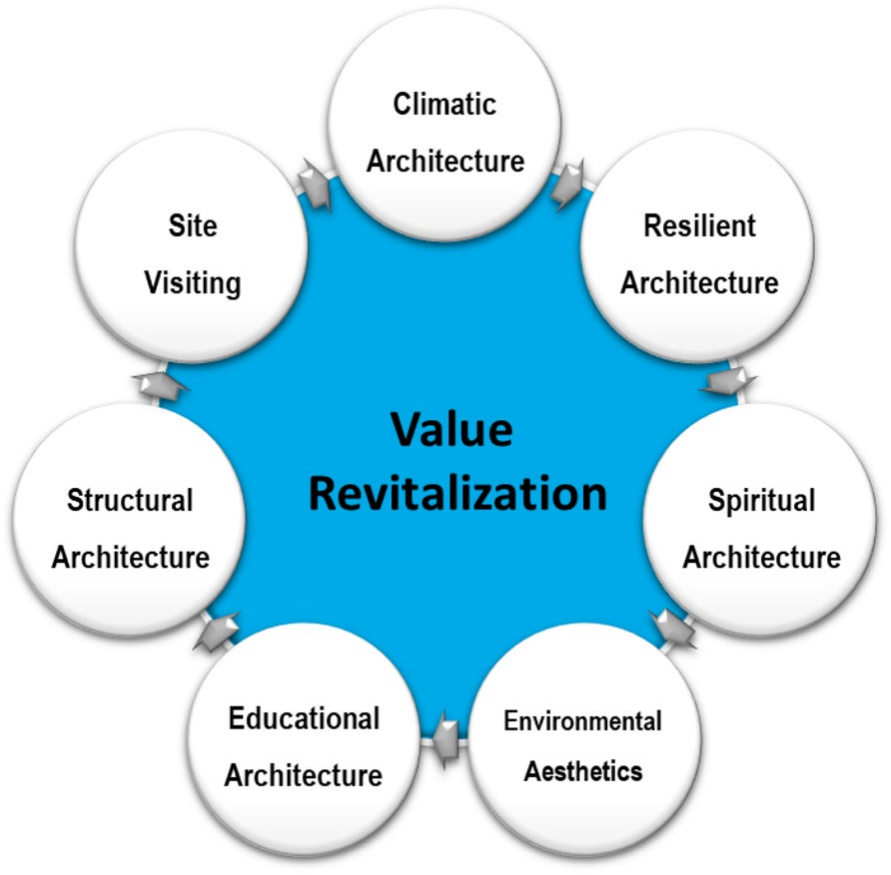
The conceptual model of value revitalization factors for Qajar religious schools in Tehran

According to the empirical study of the experts' views and the research results, to revitalize the value of Qajar religious schools in Tehran, it is required to consider seven factors (Fig. 6) including climatic architecture, resilient architecture, spiritual architecture, environmental aesthetics, educational architecture, structural architecture, and site visiting [93].

### Study strength and contribution

Since the theories and principles of religious school conservation have not been written and published by the architectural heritage experts and the Cultural Heritage, Handicrafts, and Tourism Organization of Iran, the research method used in this article is very efficient and practical to identify and classify the experts' views. Also, this research method can be useful in other architectural heritage functions for which no research has been done on the development of conservation principles. Conservation measures should be implemented based on the identified values and factors in relevant research.

### Study limitations

The main limitation of the research was the pandemic of coronavirus which led to the closure of universities and thereby making it difficult, on the one hand, to communicate with academic experts and do field studies of religious schools, on the other hand. Also, it was possible to investigate only those schools that had not been destroyed.

### Implications on practice

The innovation of this article is to provide an accurate methodological framework that helps to enrich the literature on preserving and reviving the value of historical religious schools. There are two major problems with previous research. The first is the lack of research on the conservation, preservation, and rehabilitation of these schools and the second is the lack of accurate and methodical research methods in other relevant research on these schools. One of the main steps of this article is the identification and classification of both tangible and intangible values of these schools according to the opinions of experts. The identified values will underlie all conservative measures in these schools. Also, recognizing the main factors, along with their constituent variables, in the conservation process, makes us have a deeper understanding of the hidden layers of these schools. However, to prioritize conservative measures considering financial and time constraints, it is necessary to rank the identified factors. It is expected that various priorities will be obtained for various schools according to their needs and sociocultural statuses.

The results of this article can be useful for the principals of historical religious schools to manage and maintain their schools, the Iranian Cultural Heritage Organization to conserve and revive historical schools, and for architects who want to design new religious schools.

### Future research

In this study, which is based on the Q and Delphi methods, only the experts’ mental patterns were discovered and classified. Thus, for determining the correlations between variables and factors, the future study will be a comprehensive survey through interviews with religious school users, who are students, teachers, and people.

## Conclusion

The most significant factor in the conservation and revitalization of Qajar religious schools in Tehran is to pay attention to climatic architecture. In other words, all the architectural elements of these buildings, including ornamentations, roof type, facade designs and covering, and materials (bricks) were compatible with the climate of Tehran. Moreover, in the maintenance of these schools, attention should be paid to the authenticity of their buildings, depending on the integrity and intactness of original materials.

Due to their physical resilience and unique structural systems, these buildings have been able to withstand all-natural disasters such as earthquakes, humidity, and subsidence, and remain stable. However, despite their stability, many of these schools need physical revitalization and structural strengthening. If intervention measures are not immediately taken, the schools will suffer from irreparable damage. Religious schools built during the Qajar era are still active with the same original use. Semantic resilience is the reason why these buildings have preserved their primary use. In other words, cultural and identity values in today's society are consistent with the use of these schools, and the public, in addition to students, use these buildings during prayers and do socio-religious activities in them. This is why these buildings are dynamic and have always been considered. Studies have indicated that schools prohibited for public use have been neglected due to their reduced semantic resilience, causing to diminish the need to confirm the status of such schools in society. Moreover, many have been destroyed due to the lack of public notice. The main reason for the destruction of such buildings is the urban expansion and neglecting of their proper maintenance.

Moreover, these buildings have spiritual and educational architecture. This type of architecture aims to educate and acquaint students with spiritual concepts in the language of physical architecture. To revitalize the values of these schools, it is necessary to pay attention to both spiritual and educational architecture.

From an environmental aesthetic point of view, the two parts of the courtyard from the inside of the building and created landscape (the facades, dome and minarets) by the school from the outside of the buildings are significant and should be considered in the process of conservation and revitalization.

Structural architecture is also significant in historical buildings, especially in religious schools. The structure of these schools represents the traditional structures used in Iranian architecture, possessing the vault, arch, and dome. Traditional Iranian architecture is known for its vaults and arches. People in the country have a sense of belonging to this type of structural architecture. This type of structure is beautiful and is not separate from architecture. In these buildings, the desired vault was designed and implemented according to the use of each space and required ornamentations. Thus, each vault has its own structural and geometrical identity. Further, this type of structure is economically viable because it is made of accessible local materials such as bricks, clays, and plasters.

Finally, being visited by tourists and their active presence in the religious schools will help strengthen the identity and cultural values of them. Moreover, by introducing these schools on a global scale, the ground will be provided for the value revitalization. Currently, none of these schools is allowed for public use, and also, none of them has been registered globally.

## Data Availability

All the data generated or analyzed during this study are included in this published paper.
